# Successful treatment of near-fatal pulmonary embolism and cardiac arrest in an adult patient with fulminant psittacosis-induced severe acute respiratory distress syndrome after veno-venous extracorporeal membrane oxygenation rescue: A case report and follow-up

**DOI:** 10.1016/j.heliyon.2023.e20562

**Published:** 2023-09-30

**Authors:** Song-Liu Yang, Yang Gao, Zhi-Yang Han, Xue Du, Wen Liu, Song-Gen Jin, Ying Bi, Peng-Fei Chen, Chuang-Shi Yue, Ji-Han Wu, Qi-qi Lai, Yu-jia Tang, Xin-Tong Wang, Yuan-Yuan Ji, Ming-Yan Zhao, Kai Kang, Kai-Jiang Yu

**Affiliations:** a Department of Critical Care Medicine, The First Affiliated Hospital of Harbin Medical University, Harbin 150001, Heilongjiang Province, China; b Department of Critical Care Medicine, The Sixth Affiliated Hospital of Harbin Medical University, Harbin 150028, Heilongjiang Province, China; c Institute of Critical Care Medicine, The Sino Russian Medical Research Center of Harbin Medical University, Harbin 150001, Heilongjiang Province, China; d Department of Vascular Surgery, The First Affiliated Hospital of Harbin Medical University, Harbin 150001, Heilongjiang Province, China; e Key Laboratory of Hepatosplenic Surgery, Ministry of Education, Harbin 150001, Heilongjiang Province, China; f The Cell Transplantation Key Laboratory of National Health Commission, Harbin 150001, Heilongjiang Province, China

**Keywords:** Fulminant psittacosis, Acute respiratory distress syndrome, Extracorporeal membrane oxygenation, Pulmonary embolism, Cardiac arrest, metagenomic next-generation sequencing

## Abstract

**Background:**

Veno-venous extracorporeal membrane oxygenation (ECMO) was successfully performed for the rescue of an adult patient with severe acute respiratory distress syndrome (ARDS) induced by fulminant psittacosis, and then a near-fatal pulmonary embolism (PE) and cardiac arrest (CA) of the same patient was cured through catheter-directed thrombolysis.

**Case presentation:**

A 51-year-old female patient was admitted to the hospital on September 10, 2021 due to slurred speech, weakness in lower limbs, dizziness, and nausea. Subsequently, she developed confusion and was transferred to the intensive care unit (ICU), where she received anti-shock, antibiotics, invasive mechanical ventilation (IMV), and veno-venous ECMO due to the diagnosis of severe pneumonia, severe ARDS, and septic shock based on comprehensive physical examination, laboratory tests, and imaging findings. The metagenomic next-gengeration sequencing (*m*-NGS) in the bronchoalveolar lavage fluid (BALF) suggested that the pathogen was chlamydia psittaci, so the antibiotics were adjusted to doxycycline combined with azithromycin. After withdrawal from ECMO, ultrasound (US) re-examination of the left lower limb revealed inter-muscular vein thrombosis, following which heparin was replaced by subcutaneous injection of 0.4ml enoxaparin sodium twice daily for anti-coagulation therapy. After withdrawal from IMV, the patient suffered sudden CA and successful cardiopulmonary resuscitation (CPR), and emergency pulmonary angiography (PA) was performed to show bilateral main pulmonary artery embolism. After immediate catheter-directed thrombolysis and placement of an inferior vena cava filter, the patient's condition gradually stabilized.

**Conclusions:**

Veno-venous ECMO can be successfully performed as an emergency life-saving treatment for patients with severe ARDS induced by fulminant psittacosis, and during ECMO regular examinations should be conducted to detect and manage thrombosis in time, thereby avoiding the occurrence of near-fatal PE and CA.

## 1 Introduction

*Chlamydia psittaci* is an obligate intracellular, gram-negative pathogenic bacterium that mainly infects birds, domestic poultry, and wildfowl, and can lead to highly zoonotic infectious diseases. Psittacosis is *chlamydia psittaci-*induced infection whose clinical manifestations are highly diverse and nonspecific, ranging from asymptomatic infection, influenza-like manifestations, high fever, diarrhea, and sepsis to severe pneumonia with rapid progression, acute respiratory distress syndrome (ARDS), and even life-threatening multiple organ dysfunction syndrome (MODS) [1]. Exposure to the feces and nasal discharges of infected birds or poultry is considered the main transmission route of psittacosis; however, not all infected individuals have a clear history of exposure [2]. Lack of specificity in symptoms, clinical manifestations, imaging features, and currently available conventional diagnostic approaches, as well as inadequate awareness among medical staff, can lead to increased incidence of under-diagnosed psittacosis and a serious underestimation of its public health importance [3]. Fast and accurate identification of clinical pathogens is critical in determining and accelerating subsequent targeted antibiotic therapy. Compared with traditional pathogen detection methods, metagenomic next-gengeration sequencing (mNGS) has higher sensitivity and specificity and thus represents a promising tool for rapid pathogen-specific diagnosis, especially for rare pathogens [4].

Veno-venous extracorporeal membrane oxygenation (ECMO) is a modified cardiopulmonary bypass circuit that can be routinely used to rescue patients with severe ARDS caused by different etiologies, thus gaining valuable time for the treatment of primary diseases [5]. However, thrombosis, as one of the common complications of ECMO, can sometimes lead to near-fatal pulmonary embolism (PE) and even cardiac arrest (CA) [6]. Herein, we presented a successful treatment and follow-up of near-fatal PE and CA in an adult patient with fulminant psittacosis-induced severe ARDS after veno-venous ECMO rescue.

## 2 Case presentation

A 51-year-old female patient was admitted to the Department of Neurology in The First Affiliated Hospital of Harbin Medical University on September 10, 2021 due to slurred speech, weakness in lower limbs, dizziness, and nausea. The patient had a history of cervical polyp surgery without other comorbidities. The patient had no personal or family history and no clear history of contact with birds and poultry.

Physical examination on admission showed that height, weight, body mass index (BMI), heart rate (HR), respiratory rate (RR) and blood oxygen saturation (SO_2_) were 1.6 m, 58kg, 22.65 kg/m^2^, 124 beats/min, 23 beats/min, and 88%, respectively. Furthermore, mild cyanosis of the lips, dysarthria, shortness of breath, audible wet rales in both lower lungs, and grade IV muscle strength in lower limbs were observed. Laboratory tests on admission and re-examinations during hospitalization were displayed in Table 1. Arterial blood gas analysis（ABGA）showed that pH, carbon dioxide partial pressure (pCO_2_), and oxygen partial pressure (pO_2_) were 7.554, 27.8 mmHg, and 53.6 mmHg, respectively. The calculated Acute Physiology and Chronic Health Evaluation (APACHE) II and Sequential Organ Failure Assessment (SOFA) scores were 12 and 4, respectively.

**Table 1:** Timeline of the disease course (Sep 10 to Dec 21, 2021).

Day of illness		1	2	3	4	5	6	7	8	9	10	12	13	14	15	16	17	18	19	20	21	22	23	27	28	11	74
Disease Course	Hospitalization		ICU																				Hospital discharge	Follow-up	Follow-up		
WBC ( × 10^9^/L)	12.26		9.44		17.2	17.8	10.4	10.7	7.8	8.8	9.4	12.9	9.16	8.2	8.7		9.58				10.7		5.76		7.98		6.8
NEUT% (%)	89.6		94.1		93.2	91.9	90.5	89	81.4	82.6	77.6	84.8	76.5	79.3	79.1		84.7				76.8		71.7		44.8		43.3
NEUT ( × 10^9^/L)	10.99		8.89		16.1	16.4	9.42	9.6	6.38	7.32	7.32	11.01	7	6.53	6.93		8.12				8.3		4.13		3.57		2.95
LYMPH% (%)	8.3		3.9		3	4.3	4.8	5.7	9.1	9.4	13.4	6.6	8.6	8.6	8.9		6.7				12.8		17.4		29.9		49.6
PLT ( × 10^9^/L)	146		150			87			102				167		205		217				260				369		
LYMPH ( × 10^9^/L)	1.02		0.37		0.51	0.77	0.5	0.61	0.71	0.83	1.27	0.85	0.79	0.71	0.78		0.64				1.37		1		2.39		3.37
ALT (U/L）	114.7	90.4	84.6		45.7	32.5	79.7	90.1	84.5	102.4	79.4	196.8	175.2	130.3	109.3		59.7			35.9	46		46		33.1	32.6	94
AST (U/L）	308.7	176.5	115.3		89.1	61.9	186.7	144.3	81.3	82	52.4	126.7	89.1	52.5	42.5		23.9			15.4	29		29		20.4	30.8	48
PCT (ng/mL)	3.72		2.12		8.32	5.42	2.43		0.52	0.34		0.15		0.09	0.14					0.39							
FIB (g/L)	8.73		7.4		5.5	5.53	3.33	2.67	2.24	2.18	2.24	3.74	4.71	3.84	5.67		4.92		3.93	3.9	3	3.24	3		2.66	2.53	3.18
D-dimer (μg/mL)	8.1		14.88		15.38	6.5	4.57	4.77	5.15	5.92	4.5	3.89	7.81	6.98	7.74		9.73		9.26	8.51	3.29	2.91	3.29		2.87	1.65	0.36
Oxygen therapy	Nasal catheter oxygen therapy		HFNC	IMV + ECMO									IMV				HFNC		IMV		HFNC			Nasal catheter oxygen therapy			
ECMO gas				3	3	3	3	4	4	4	3	3	3														
folw (L/min)																											
ECMO blood flow (L/min)				3	3	3	3	3.13	3	3	3	3	3														
ECMO Fi0_2_				100%	100%	100%	100%	90%	90%	90%	90%	60%	0														
ACT（sec）				187		194			210																		
IMV FiO_2_	4L/min	4L/min	50%	100%	40%	40%	40%	40%	40%	40%	40%	40%	40%	40%	40%		50%	50%	100%	40%	40%	40%	40%	4L/min			
PEEP (cmH_2_O)				18	16	15	15	14	12	12	10	10	8	6	6					5	5						
PO_2_ (mmHg)	53.6		68.6	56.1	87.7	108	129	125	124	106		127	122		114		86.4		13.1	138	121			114			123
PCO2 (mmHg)	27.8		28.5	26.2	26.4	47.8	36.4	38.3	38.7	36		38.6	43.9				38.6		45.4								44.6
Lac（mmol/L）	0.9		1.1	1.1	1.5	3.3	3.2		1.5		1.7	1	0.8	1	0.7	0.6	0.7	1	0.6	0.6	1.6	0.4	1.2	1			0.7
OI (mmHg)	144.9		137.2										305		285		172.8		13.1	345	302.5			308.1			333
Antibiotics	piperacillin-tazobactam		cefoperazone/sulbactam combined						doxycycline combined						erythromycin combined												
			with levofloxacin						with azithromycin						with levofloxacin												

**Fig. 1:**
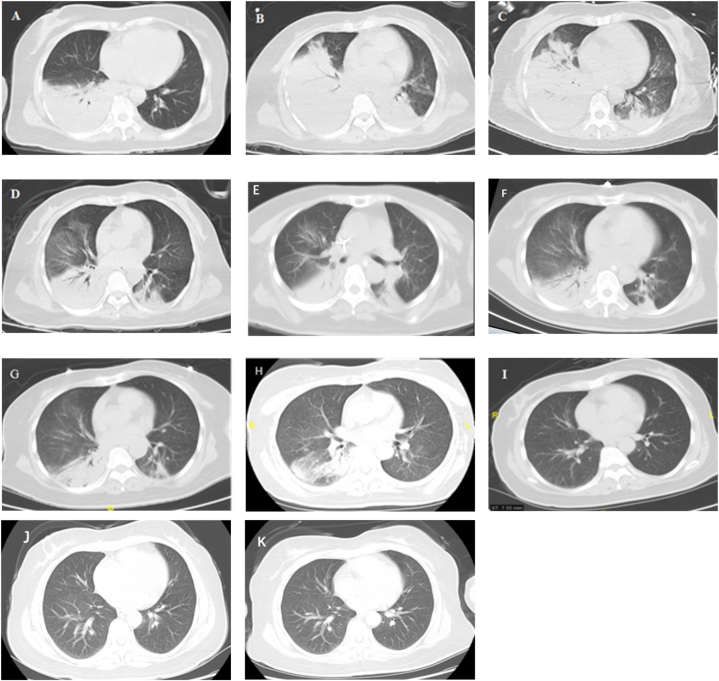
**Lung computed tomography of the patient during hospitalization and follow-up. (A)** On admission; **(B)** On the3^rd^ day after admission; **(C)** On the 5th day after admission; **(D)** On the 14th day after admission; **(E)** On the 17th day after admission; **(F)** On the 20th day after admission; **(G)** On the 25th day after admission; **(H)** On the 11th day of follow-up after discharge; **(I)** On the 74th day of follow-up after discharge; **(J)** On the 313th day of follow-up after discharge; **(K)** On the 502nd day of follow-up after discharge.

Magnetic resonance imaging (MRI) of the brain on admission revealed a reversible lesion of the corpus callosum. The initial lung computed tomography (CT) indicated extensive patchy and consolidation shadows in the right lower lobe and left pleural hypertrophy. Lung CT during hospitalization and follow-up were displayed in Fig. 1(A-K).

**Fig. 2:**
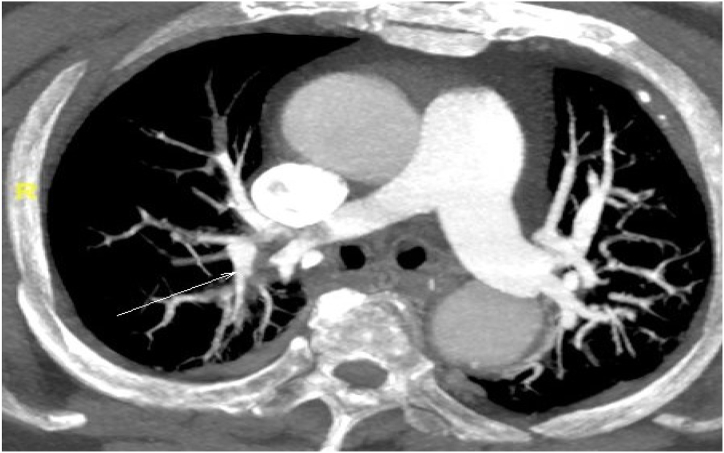
Computed tomographic pulmonary angiography: bilateral main pulmonary artery embolism.

Immediately after admission, the patient was administered nasal catheter oxygen therapy and piperacillin-tazobactam for anti-infection therapy. On the 2nd day after admission, the patient developed a fever with a maximum temperature of 39.1 °C, accompanied by lethargy and confusion, and thus was transferred to intensive care unit (ICU), where she received high-flow nasal cannula (HFNC) oxygen therapy, and antibiotics were adjusted to cefoperazone/sulbactam combined with levofloxacin. On the 3rd day after admission, the re-examination of lung CT showed a significant increase in lesion area, and blood pressure decreased. Other routine imaging examinations, including ultrasound (US) of both lower limbs, showed no abnormality in ICU. Comprehensive treatment measures, including goal-directed fluid resuscitation, vasoactive drug infusion, oral endotracheal intubation, invasive mechanical ventilation (IMV) with low tidal volume (4–6ml/kg), optimal positive end-expiratory pressure (PEEP), lung recruitment, deep analgesia and sedation combined with neuromuscular blockers, and prone position, were implemented. The patient was not treated with corticosteroids due to the possibility of progressive exacerbation of the infection. Flexible fiberoptic bronchoscopy (FFB) revealed that the bronchial openings of some lobes and segments in the right lung were blocked by a small amount of yellow mucous sputum, and the airway mucosa was congested. Bronchoalveolar lavage (BAL) was subsequently performed, and samples were taken for mNGS. After oxygenation index (OI) < 60 mmHg for 12 consecutive hours without improvement, the left femoral vein and right internal jugular vein were taken for veno-venous ECMO with heparin anti-coagulation. Activated clotting time (ACT) of whole blood was monitored every 4 hours to guide heparin administration and maintained within the optimal target range of 180–220 seconds whenever possible. During veno-venous ECMO, goal-directed fluid management, lung-protective ventilation strategies, deep analgesia and sedation combined with neuromuscular blockers, and prone position were continued. On the 8th day after admission, the mNGS in the bronchoalveolar lavage fluid (BALF) revealed that the pathogen was chlamydia psittaci, so the antibiotics were adjusted to doxycycline combined with azithromycin. On the 13th day after admission, ECMO was withdrawn after 10 days of clinical application, and the patient's condition improved with an OI of 305 mmHg. US re-examination of the left lower limb revealed inter-muscular vein thrombosis, so heparin was replaced by subcutaneous injection of 0.4ml enoxaparin sodium twice daily for anti-coagulation therapy. Considering that the extensive rash may be related to doxycycline, the antibiotics were adjusted to erythromycin combined with levofloxacin.On the 17th day after admission, the patient was weaned from IMV. On the 19th day after admission, the patients suffered sudden CA, and the autonomous heart rhythm was restored after about 2 minutes of successful cardiopulmonary resuscitation (CPR). Emergency pulmonary angiography (PA) showed bilateral main pulmonary artery embolism (Fig. 2**)**, and the patient's condition gradually stabilized after immediate catheter-directed thrombolysis with 300,000 units of urokinase and placement of an inferior vena cava filter. On the 21st day after admission, the patient was fully conscious with stable vital signs, so she was weaned from IMV again. On the 28th after admission, the patient fully recovered and was discharged. On the 11th day of follow-up after discharge, lung CT showed pneumonia in the left lower lobe and right lung and mild pleural hypertrophy on both sides. On the 74th day of follow-up after discharge, the inferior vena cava filter was removed. The re-examined lung CT indicated interstitial changes in the right lower lobe, while pulmonary function tests, OI, and post-traumatic stress disorder (PTSD) checklist-Civilian (PCL-C) score returned to normal. On the 313th day of follow-up after discharge, lung CT revealed mild interstitial changes in the right lower lobe, ground-glass nodules in the right upper lobe, and nodules in the left upper lobe. On the 502nd day of follow-up after discharge, lung CT showed small nodules in the right upper lobe, and the rest completely returned to normal.

## 3 Discussion

*Chlamydia psittaci* is named after its host. Fever, chills, dyspnoea, headache, myalgia, non-productive cough, and expectoration are the most common and non-specific clinical symptoms of psittacosis, however this condition can also affect all organ systems, not just the respiratory system [7]. In the present study, the patient initially presented with atypical symptoms, pointing to the nerve system, thus posing a significant difficulty in making an accurate diagnosis in the early stages of the disease. Although the incidence of this condition is low, the possibility of psittacosis should be considered for unexplained community acquired pneumonia (CAP), where bird exposure may serve as a clue in diagnosis rather than a decisive factor, as exposure to aerosols or feces in the environment can also lead to infection [8].

The features of lung CT found in patients with pneumonia caused by *chlamydia psittaci* include consolidation, ground-glass opacity, multiple lobar distributions, and pleural thickening, with air bronchogram in some patients, which also lack specificity compared with pneumonia caused by other common pathogens [9]. In addition, patients with psittacosis respond well to prompt organ support and targeted antibiotics, and therefore veno-venous ECMO are rarely needed as the last line of defence for rescue in clinical practice [9,10].

The intracellular lifestyle of *chlamydia psittaci* makes it more complicated and difficult to obtain an accurate diagnosis [11]. mNGS is essentially a culture-free and hypothesis-free nucleic acid test that can identify all pathogens with known genomic sequences, thereby greatly expanding the clinical capacity of pathogens detection [12]. By amplifying and sequencing the nucleic acids of pathogens, mNGS can quickly and accurately identify multiple pathogens from all kinds of samples for disease diagnosis, especially for rare pathogens with limitations in conventional diagnostic approaches [13]. Due to the lack of specific symptoms, clinical manifestations, imaging features, and available conventional diagnostic approaches for psittacosis in clinical practice, mNGS has the potential to identify it at an early stage, following which targeted antibiotics can be accordingly adjusted to improve prognosis [9,14]. In addition, it is equally important to raise awareness of psittacosis among medical staff.

Even with low tidal volume, optimal PEEP, deep analgesia and sedation combined with neuromuscular blockers, lung recruitment, prone position, and restrictive fluid management during IMV, it is sometimes impossible to reverse the refractory hypoxemia and severe respiratory acidosis induced by severe ARDS and make the body's oxygen supply and consumption to re-balance at the pathological level. As a result, in-hospital mortality of patients with severe ARDS remains unacceptably high, ranging from 26% to 60% [15,16]. After OI is < 60 mmHg for 12 consecutive hours without any improvement, veno-venous ECMO should be initiated in order to implement lung-protective ventilation strategies and put the lungs on temporary rest to avoid exacerbating existing lung injury, provide gas exchange, and improve systemic perfusion and metabolism. It has been confirmed that veno-venous ECMO can improve survival and outcomes in patients with severe ARDS [16,17]. In this case, when conventional CPR failed to achieve the desired results, timely utilization of veno-arterial ECMO for extracorporeal CPR (ECPR) should be considered to stabilize the patient's vital signs, augment perfusion to vital end-organs, gain valuable time for finding and reversing the aetiologies of sudden CA and improve survival [17,18]. On the 313th day of follow-up after discharge, lung CT of the patient revealed mild interstitial changes in the right lower lobe, ground-glass nodules in the right upper lobe, and nodules in the left upper lobe, suggesting that more time is needed to repair the lung injury caused by fulminant psittacosis-induced severe ARDS.

Although standard medical and physical measures and ongoing monitoring are taken during ECMO to prevent deep venous thrombosis (DVT), it can still occur and cause near-fatal PE and CA, which might be primarily related to the mechanical damage from ECMO catheterization, in addition to extracorporeal circulation and sepsis-induced coagulopathy. Vessel wall injury is one of the high-risk factors for thrombosis. For patients suspected of PE, emergency interventional angiography can not only clarify the causes of disease deterioration, but also precisely deliver thrombolytic drugs to the local area, relieve vascular obstruction, and improve clinical symptoms and outcomes through catheter-directed thrombolysis [18,19]. Other treatments for PE include catheter-directed clot extraction, systemic thrombolysis, open operation with clot extraction, or veno-arterial ECMO and heparin anti-coagulation waiting for spontaneous thrombolysis.

## 4 Conclusion

The possibility of psittacosis should be considered for unexplained CAP, where bird exposure history may serve as a clue in diagnosis rather than a decisive factor. Compared with traditional pathogen detection methods, mNGS has higher sensitivity and specificity and thus represents a promising tool for rapid pathogen-specific diagnosis, especially for rare pathogens, which is of great clinical significance for the adjustment of antibiotics. Veno-venous ECMO can be successfully performed to rescue patients with severe ARDS induced by fulminant psittacosis, thus gaining valuable time for treating primary disease. During ECMO, regular examinations should be conducted to detect and manage thrombosis in time, thereby avoiding the occurrence of near-fatal PE and CA. For patients suspected of PE, emergency interventional angiography contributes to clarify the causes of disease deterioration, following which appropriate treatments can be provided. Last, a long time of nearly 1 year is necessary to repair the lung injury caused by fulminant psittacosis-induced severe ARDS from the follow-up of our case.

## 5 Consent for publication

Informed consent was obtained from the patient's guardian for the publication of all images, clinical data and other data included in the manuscript.

## Data availability statement

Data included in article/supp. material/referenced in article.

## CRediT authorship contribution statement

**Song-Liu Yang:** Writing – review & editing, Writing – original draft. **Yang Gao:** Writing – review & editing, Writing – original draft. **Zhi-Yang Han:** Writing – original draft, Funding acquisition, Data curation. **Xue Du:** Data curation. **Wen Liu:** Investigation, Data curation. **Song-Gen Jin:** Investigation, Data curation. **Ying Bi:** Data curation. **Peng-Fei Chen:** Data curation. **Chuang-Shi Yue:** Data curation. **Ji-Han Wu:** Investigation, Data curation. **Qi-qi Lai:** Investigation. **Yu-jia Tang:** Investigation. **Xin-Tong Wang:** Methodology. **Yuan-Yuan Ji:** Data curation, Conceptualization. **Ming-Yan Zhao:** Investigation, Funding acquisition. **Kai Kang:** Investigation, Data curation. **Kai-Jiang Yu:** Investigation, Formal analysis, Data curation.

## Declaration of competing interest

The authors declare that they have no known competing financial interests or personal relationships that could have appeared to influence the work reported in this paper.
