# Supplementary material for: Successful treatment of near-fatal pulmonary embolism and cardiac arrest in an adult patient with fulminant psittacosis-induced severe acute respiratory distress syndrome after veno-venous extracorporeal membrane oxygenation rescue: A case report and follow-up
Source: Heliyon. 2023 Sep 30;9(10):e20562. doi: 10.1016/j.heliyon.2023.e20562 (PMC10568334; doi:10.1016/j.heliyon.2023.e20562)
Supplement: Multimedia component 1 [file mmc1.pdf]

“晶” 准 出 击 · 寻 觅 病 原

晶觅原™

病原体宏基因组学
快速检测报告

GENTALKER
FOR
HEALTH

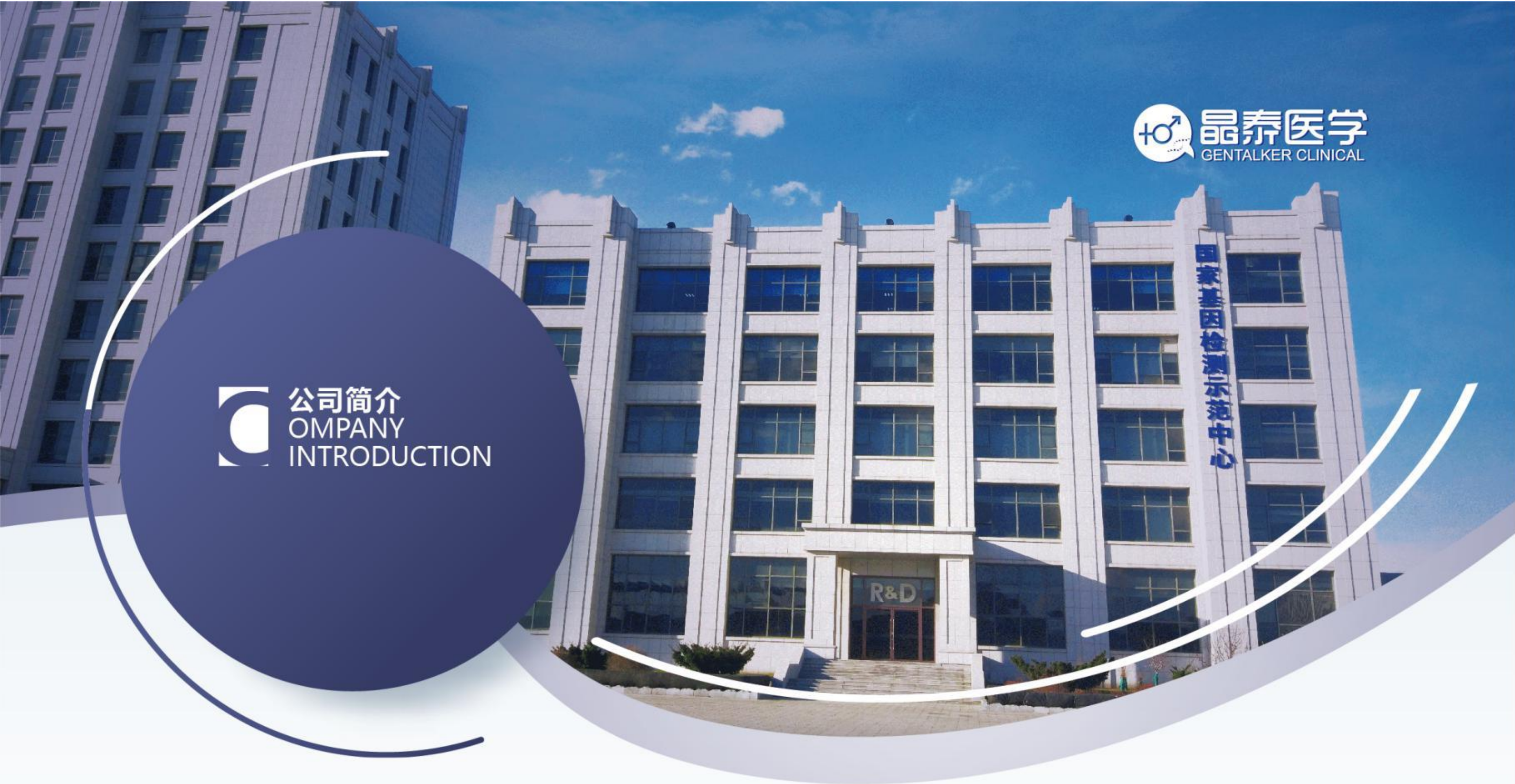

公司简介
COMPANY INTRODUCTION

大连晶泰医学检验实验室有限公司

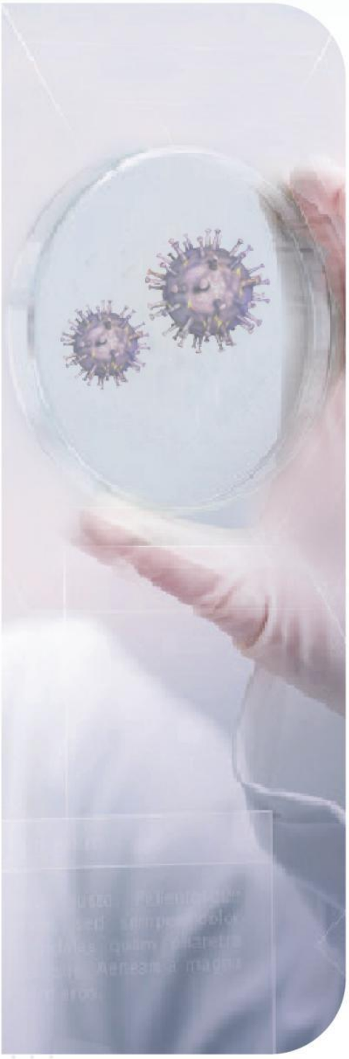

晶泰医学是以二代测序技术为核心，集研发、生产、科研服务和医学检验于一体的医学检验实验室，具备临床基因扩增检验资质、设立艾滋病初筛实验室、生物安全二级实验室。2017 年经国家发改委批准成立国家基因检测示范中心。2019 年获批成为高新技术企业。2020 年经辽宁省卫健委指定、临检中心审核，成为国内首批新冠病毒核酸检测实验室。

晶泰医学是中国最早深耕于 mNGS 病原感染诊断的实验室之一，也是国家十三五国家重点专项课题中唯一的病原宏基因组测序实验室。晶泰医学核心团队在基因测序领域经验丰富，曾参与埃博拉病毒、SARS 病毒以及新冠病毒溯源分析工作。作为mNGS病原诊断专家共识参与单位，晶泰医学拥有数十项自主知识产权发明专利，国外高水平专业期刊发表 SCI 论文百余篇。

晶泰医学致力于为医学专家提供全覆盖的个体化医学解决方案，成为精准医学行业的驱动者。

病原体宏基因组学快速检测报告

基本信息

姓名:	王凤华	性别:	女	年龄:	51 岁
身份证号:				联系方式:	
临床诊断结果:	重症肺炎				
既往病史:	--				
抗感染用药史:	--				

样本信息

样本编号:	T210602179	样本采集日期:	2021-09-14
样本类型:	肺泡灌洗液	样本接收日期:	2021-09-15
样本状态:	合格	检测完成日期:	2021-09-18

检测者：于红　　审核者：王春红　　报告日期:2021-09-18

大连晶泰医学检验实验室有限公司 -报告专用章-

检测项目

本项目将对委托送检样本中的 DNA/RNA 进行高通量测序，将获得的数据与病原微生物数据库进行比对分析，获得疑似致病微生物相关信息。本项目可检测范围包括病毒、细菌、真菌、寄生虫等基因组序列已知的微生物，并提供全面深入的报告参数，辅助临床快速诊断感染病原，从而辅助临床医生进行分析判断。

说明：

1. 本报告仅供医生参考；
2. 检测对于样本内 DNA/RNA 进行检测，不排除有其他低浓度病原体存在的可能性；
3. 检测结果仅对本样本负责，如有疑问，请拨打遗传咨询电话：4000-768-568。

防伪验证码
查询报告真伪

检测结果

1. 检出病毒列表

中文名	英文名/拉丁名	reads 数量	rpm
**EB 病毒**	*Human gammaherpesvirus 4*	23	0.821

注: rpm 为标准化到每百万 reads 检测到的检测值，rpm 值越大，对应的病原拷贝数越多。

2. 检出细菌列表

中文名	英文名/拉丁名	reads 数量	rpm
-	*Chlamydia abortus*	10696	381.728
**鹦鹉热衣原体**	*Chlamydia psittaci*	686	24.483

注: rpm 为标准化到每百万 reads 检测到的检测值，rpm 值越大，对应的病原拷贝数越多。

3. 检出真菌列表

中文名	英文名/拉丁名	reads 数量	rpm
**未检出真菌**			

注: rpm 为标准化到每百万 reads 检测到的检测值，rpm 值越大，对应的病原拷贝数越多。

4. 检出寄生虫列表

中文名	英文名/拉丁名	reads 数量	rpm
**未检出寄生虫**			

注: rpm 为标准化到每百万 reads 检测到的检测值，rpm 值越大，对应的病原拷贝数越多。

5. 耐药基因检测结果

耐药基因分类	耐药基因	耐药种类	覆盖度	reads 数量
**未检出耐药基因**				

结果说明：

综合本例样本中的 rpm 值等参数对检出的微生物进行测序，如上表所示，综合排名靠前的微生物在样本中的相对含量较高。

据以上列表，与正常人相比，*Chlamydia abortus* 含量最高。请医生根据临床症状做具体判断。

现将表中所列物种的致病信息进行简介：

1.鹦鹉热衣原体介绍

鹦鹉热衣原体为革兰氏阴性，光学显微镜下可见，最初认为鹦鹉是该病原体的宿主而将其引起的疾病称为鹦鹉热，又名鸟疫。鹦鹉热衣原体感染(鸟疫)是人类、鸟类及一些哺乳动物均易感鹦鹉热衣原体所引起的自然疫源性衣原体病人类感染主要是由排菌鸟及其污染物引起的，故是典型的动物源性传染病。通常表现为高热、恶寒、头痛肌痛、咳嗽和肺部浸润性病变等特征一般症状颇似感冒，但多数患者都出现肺炎。

2.EB 病毒介绍

EB 病毒（人类疱疹病毒 4 型）是疱疹病毒科的一种包膜病毒。它有一个二十面体衣壳（球形）的形式。在成人中，EBV 感染通常会引起腺热。EBV 也与肿瘤的形成有关。 主要的传播途径是直接或间接接触受污染的人或物体。

附录

疑似背景微生物列表，不排除该列表内微生物引起感染的可能。

中文名	英文名/拉丁名	reads 数量	rpm
肺炎克雷伯菌	*Klebsiella pneumoniae*	48	1.713
人型支原体	*Mycoplasma hominis*	3	0.107
肺炎链球菌	*Streptococcus pneumoniae*	2	0.071

注: rpm 为标准化到每百万 reads 检测到的检测值，rpm 值越大，对应的病原拷贝数越多。

相关说明：

1.肺炎克雷伯菌介绍

肺炎克雷伯菌是一种兼性厌氧型，革兰氏阴性杆状细菌，属于肠杆菌科。最常见的肺炎克雷伯菌感染包括下呼吸道感染和导管相关性尿路感染。由于肺炎克雷伯菌产生 β—内酰胺酶，对青霉素和氨苄青霉素的抗药性越来越强。此外，这种细菌属于 ESBL（超广谱 β-内酰胺酶)产生菌株（ESBL=extended-spectrum beta-lactamase），因此对具有广泛活动范围的抗生素具有越来越多的耐药性，如头孢菌素或头孢他啶。主要的传播途径是直接或间接接触受污染的人或物体。

2.人型支原体介绍

人型支原体是支原体的一种，它存在于泌尿系和生殖器中，可以引起泌尿系的感染和生殖器的炎症。成人主要通过性接触传播，新生儿则由母亲生殖道分娩时感染。成人男性的感染部位在尿道粘膜，女性感染部位在宫颈。新生儿主要引起结膜炎和肺炎。

3.肺炎链球菌介绍

肺炎链球菌是一种需氧革兰氏阳性细菌。它的形状是椭圆形的，可以呈双链或短链形式。肺炎链球菌属于链球菌科的家族。 它经常引起肺炎和支气管肺炎，也可能导致脑膜炎或败血症。近年来，它的抗性增加了，特别是对青霉素和大环内酯类。在南欧国家，耐药性往往特别高。传播主要通过空气中的飞沫或微粒传播。

参考文献

[1] Jia et al. 2017. CARD 2017: expansion and model-centric curation of the Comprehensive Antibiotic Resistance Database. Nucleic Acids Research, 45, D566-573.

产品简介

晶觅原 ™ 基于行业领先的 ILLumina 测序平台，对样本中所有微生物进行检测，通过 mNGS 宏基因组分析策略，与晶泰医学自建专业病原微生物数据库进行物种对比，结合晶泰 ICSQ™ 物种鉴定智能分析算法，准确获取病原微生物**种属信息及细菌耐药信息**。辅助临床快速、精准制定抗感染治疗方案。

检测范围
18012

细菌9102种
真菌2019种
病毒6065种
寄生虫301种
衣原体142种
分枝杆菌147种
立克次体105种
支原体131种

核心技术

更快

DNA+RNA 并建库技术
ICSQ™ 物种鉴定智能算法
36-72 小时交付报告

更全

18000+ 自建病原数据库
病原信息**全面注释**
区分**致病菌、背景菌**

更准

甲基化去宿主**专利技术**
样品处理**差异化 SOP**
保证各样本测序量，符合专家共识要求

更强

国内 mNGS **先行者**
mNGS 专家共识**参与人**
国家十三五课题**承担单位**

电话：400 0768 568
网址：www.gentalker.com
地址：辽宁省大连经济技术开发区金七路 9-2 号

晶泰医学
GENTALKER CLINICAL
